# Advancing clinical research in the Spanish primary care service: Challenges, opportunities, and lessons from other European models

**DOI:** 10.1080/13814788.2025.2589564

**Published:** 2025-12-15

**Authors:** Jose Cárdenas-Quesada, Ángela Remesal-Doblado, Vanesa Garrido-Rodríguez, M. Isabel Lucena, Leovigildo Ginel-Mendoza, Salvador León Cárdenas-Viedma, Judith Sanabria-Cabrera

**Affiliations:** ^a^ Servicio de Farmacología Clínica, Unidad de Investigación Clínica y Ensayos Clínicos, Instituto de Investigación Biomédica de Málaga y Plataforma en Nanomedicina (IBIMA Plataforma BIONAND), Hospital Universitario Virgen de la Victoria, Málaga, España; ^b^ Departamento de Farmacología, Facultad de Medicina, Universidad de Málaga, Málaga, España; ^c^ Plataforma de Investigación Clínica, SCReN, ISCIII, Madrid, España; ^d^ Centro de Investigación Biomédica en Red Enfermedades Hepáticas y Digestivas (CIBERehd), Madrid, Spain; ^e^ Medicina Familiar y Comunitaria, Centro de Salud Ciudad Jardín, Málaga, España; ^f^ Medicina Familiar y Comunitaria, Centro de Salud de Cartuja, Granada, España

**Keywords:** Primary care research, health-system integration, real-world evidence, research networks, digital tools, pragmatic trials

## Abstract

**Background:**

As the first point of contact with the healthcare system, primary care (PC) provides a unique setting for clinical research, enabling longitudinal follow-up, early disease detection, and study of prevalent illnesses. In Spain, however, PC research remains largely untapped, despite the country’s leadership in overall clinical trial initiations within the European Union.

**Objectives:**

To analyse the main challenges and opportunities of PC research in Spain and to draw lessons from successful European models to inform future priorities.

**Methods:**

This opinion paper presents a narrative, cross-country comparative synthesis (1993–2025) of peer-reviewed and gray literature.

**Results:**

European models demonstrate how interoperable data, dedicated infrastructure, and protected research time enable pragmatic and decentralised trials with high external validity. In Spain, major barriers include heavy workloads, limited research training, uneven infrastructure, and regulatory hurdles. Nevertheless, opportunities are emerging through digital platforms, interoperable registries, remote monitoring, and the integration of patient-reported outcomes (PROs).

**Conclusion:**

Spain can unlock the untapped research capacity of PC by prioritising four reforms: (1) interoperable registries across care levels; (2) protected time and support roles for PC professionals; (3) targeted, sustained funding and minimum viable infrastructure; and (4) streamlined ethical/regulatory pathways for multicentre and decentralised trials. These steps would align research with daily practice, enhance inclusivity and equity, and strengthen PC as a hub for patient-centered innovation.

## Introduction

Since its establishment in 1978, the Family and Community Medicine specialty in Spain has been essential in ensuring accessible healthcare services for the population. Integrating research advances into clinical practice has become increasingly important to improve patient care, uphold high standards of care and foster innovation.

Recent assessments by the World Health Organisation confirm that long-standing structural weaknesses in clinical research continue to persist within Spain’s primary health care system: chronic underfunding, workforce shortages, heavy workloads limiting research time, uneven governance/coordination, and insufficient support for research infrastructure and capacity [1]. Excessive workloads, limited methodological expertise, and scarce financial or curricular incentives all preclude primary care (PC) capacity.

Despite being reported 3,042 PC facilities versus 751 hospitals in Spain [2], less than 8% of clinical studies included at least one PC centre [3]. This imbalance explained partly the predominance of studies on pathologies that, due to their nature, severity, inclusion criteria or recruitment capacity, are addressed in hospitals, concentrating research in that setting to the detriment of PC.

This opinion paper analyses primary care’s research potential and how clinical research could benefit from its ability to address prevalent health problems more inclusively, considering the heterogeneity in a patient- and community-centered perspective. To support this analysis, we conducted a narrative, cross-country comparative synthesis (1993–2025) of peer-reviewed and gray literature, organising the comparison around three domains relevant to research-system capacity: (1) research infrastructure and governance; (2) funding mechanisms and protected research time; and (3) training and professional development (Box 1).

## Successful models and lessons from European primary care research

Several European countries are promoting translational research, demonstrating the progress achievable through well-established national data infrastructures, dedicated networks and sustained public funding.

The Nordic countries offer the strongest models, while large systems, such as the United Kingdom and Germany are implementing ambitious, evolving strategies. These approaches have distinct strengths and challenges and may offer relevant lessons for Spain. One compelling example is found in the Scandinavian countries, with Norway among the best. Norway stands out for integrating the Norwegian Patient Registry and the Primary Health Care Registry, linking hospital and PC nationwide. This unification has enabled multiple cohort studies using unique personal identifiers to enable detailed patient trajectories and comprehensive population studies [4].

Denmark exemplifies the effectiveness of fully integrated registries. The integration of nationwide prescription, laboratory, hospital and general practitioner databases, allows real-time pharmacoepidemiology studies and cluster trials on multimorbidity, with efficient recruitment directly from registries [5].

Jointly, the Nordic countries (Denmark, Norway, Sweden, Finland, and Iceland) have developed an interoperable environment for registry-based research. Personal identification numbers enable integration of health and demographic data within and across nations. The Nordic Medico-Statistical Committee (NOMESCO) supports harmonisation, promotes multinational studies, and addresses regulatory barriers, positioning the region as a global leader in registry-based research, demonstrating that institutional support, interoperability and access are key to success [6].

The United Kingdom explicitly recognise research as part of healthcare activities. The National Institute for Health and Care Research (NIHR) funds PC research infrastructure and provides access to electronic health records via the Clinical Practice Research Datalink (CPRD), covering 60 million patients. This has enabled trials such as DaRe2THINK within the National Health Service (NHS) using routine, Electronic Health Records (EHR)-based recruitment and data collection from the CPRD [7]. However, the NIHR Clinical Research Network Primary Care Strategy also highlights ongoing systemic challenges: limited academic pathways for general practitioners, insufficient time and funding, and the need for a cultural shift to fully integrate PC into national research [8].

German’s DESAM-ForNet initiative (launched in 2020) is PC research by accrediting over 1360 PC centres (target 1732 by 2025) and promoting ‘from practice, for practice’ research (DESAM-ForNet Consortium) [9]. Combining local recruitment and training with national coordination, it ensures patient involvement and uses a federated digital infrastructure for feasibility queries, standardised data collection, multi-site recruitment. This model aims to embed PC research into academic medicine and routine ambulatory care, enhancing external validity and implementation.

Key success factors include national and federated electronic registries, formal PC research networks with protected research time for general practitioners and support staff, and strategic public investment prioritising PC research. Together, these elements have enabled large clinical trials with superior external validity, and observational studies to be set up directly within European communities, offering a framework that could inform ongoing reforms in Spain.

## Clinical research opportunities in primary care in Spain

According to the latest EFPIA–IQVIA report, Spain recorded the highest number of new commercial clinical trial initiations in the European Union in 2023 (485, versus 417 in Germany) [10]. Despite this leadership position, PC settings remain under-represented: only 328 initiated clinical trials involved at least one PC centre, accounting for just 7.4% of all industry-sponsored trials during 2020 to 2024 (4456 in total), according to Farmaindustria reports [3]. Yet, PC offers unique advantages compared to hospital-based research, providing the routine, population-wide context in which most prescribing and care decisions occur, making it the most suitable real-world setting to evaluate interventions.

First, PC allows the inclusion of patients with multimorbidity, various risk factors or vulnerable status [11] (e.g. those predisposed to disease, pregnant women, paediatric patients or the elderly), usually excluded from clinical trials, which generates more representative cohorts and improves external validity. Such inclusiveness enhances equity, mitigates bias, and brings research closer to everyday patients [12]. In fact, the participation of vulnerable population is increasingly prioritised, reflecting demands from major regulatory agencies for stronger evidence in clinical trial settings [13].

The so-called pragmatic trials, which seek to evaluate interventions in real-world settings, have demonstrated to provide more applicable and generalisable evidence to the population, as shown by the pioneer GISSI-2 myocardial infarction trials [14]. In Spain, the DAPA-TAVI clinical trial is an excellent example of pragmatic research that could be extended to a PC or shared-care settings [15]. This study assessed the effect of dapagliflozin, added to standard post-discharge therapy, on clinical outcomes in patients undergoing transcatheter aortic valve implantation (TAVI). Although performed in tertiary care, it involves patient long-term follow-up and the inclusion of a vulnerable population group, often excluded from clinical trials in the field.

The flexible structure of the system and its inherent adaptability facilitates the evaluation of new study designs and the investigation of gaps in types of care (preventive, acute, chronic, and palliative) and interventions (pharmacological, behavioural, and structural models of patient care) in adaptive clinical trials [11,16]. Indeed, PC has unique research priorities that differ from tertiary care, including addressing psychosocial and organisational questions: doctor–patient communication, shared decision-making, management of functional symptoms, care-team well-being, innovation uptake or clinic workflows are topics often best studied with qualitative, mixed‑methods, action‐research or implementation designs [17]. For example, surveys of general practitioners consistently show high interest in issues like polypharmacy, patient safety and care coordination [18].

Furthermore, certain studies are ideally suited to PC, such as large-scale screenings for prevalent diseases, preventive initiatives, research in early stages and those requiring large patient recruitment. Employing strategies that integrate both classical and pragmatic trial designs and conducting research within PC units will help ensure the real applicability of results [19] (Table 1).

**Table 1.** Challenges and proposals for improving clinical research opportunities in primary care.

Identified Barriers in Spanish Primary Care	Lessons from Established European Networks	Proposed Actions for Spain
Heavy workload and lack of protected research time	**UK:** Research is integrated into clinical roles with paid clinical–academic contracts and performance-based funding. **Denmark and Norway**: National grants and dedicated fellowships allow GPs to reduce clinical time for research.	**Recognise research time** within statutory schedules (Law 17/2022) and expand mixed **clinical–academic contracts** across all regions. Launch intensification programmes to relieve clinical burden for active researchers.
Deficient infrastructure and lack of research-ready facilities	**Germany**: DESAM-ForNet certifies ‘research-ready’ practices and provides a central digital platform for data and coordination. **Norway:** PraksisNett uses local Snow devices for secure, real-time research integration at the point of care.	Channel the ‘Plan de Acción de Atención Primaria y Comunitaria 2023′ (€493 M) towards **upgrading health centre infrastructure and IT systems** to make them research-ready.
Limited funding and imbalance compared with hospital-based research	**UK and Germany:** Dedicated, long-term public funding (NIHR, BMBF) supports research in primary care with separate budgets from service delivery.	Increase ISCIII-AES investment; issue **targeted calls** for primary care; and **earmark funding** in the 2024–2027 national R&D strategy and regional initiatives.
Fragmented collaboration between care levels	**Nordic countries:** Use of interoperable health registries enables linkage between primary and hospital care for longitudinal research.	Establish **joint research networks** and interoperable **data platforms** connecting primary and hospital care for chronic and translational studies.
Limited engagement with industry and external partners	**UK and Denmark:** Co-funded public–private partnerships, with formalised frameworks for collaboration and reinvestment in primary care research.	Develop **formal frameworks for public–private collaboration**, including co-funded consortia, digital platforms, and regional academic–industrial clusters.
Opportunities		
Addressing **prevalent and pluripathological** diseases First patient contact and **longitudinal follow-up** Access to **heterogeneous** and historically **underrepresented populations** in clinical trials Implementation of **preventive measures** based on early detection Conducting pragmatic clinical trials to evaluate **effectiveness in real** clinical practice **conditions** **Patient-centred** research, from the measurement of reported outcomes (PROs) to participation in study design Incorporation of **digital tools and technological innovations**, to facilitate remote monitoring and inclusion in routine clinical practice Addressing **psychosocial and psycho-emotional domains,** including communication, shared decision-making, functional symptoms, mental health, and social determinants		

In this context, PC provides an ideal environment for the digital transformation of health research. Numerous ongoing initiatives evaluate the effectiveness of medical devices and digital tools in facilitating remote clinical monitoring and the collecting of continuous, high-quality clinical data directly from patients (known as patient-reported outcomes, PROs). Among these, electronic patient-reported outcomes (ePROs) allow continuous digital monitoring of adverse effects, complications, and functional status, often capturing subjective symptoms more accurately than clinician reports. In decentralised trial models, ePROs, combined with telemedicine and wearables, enable real-time safety monitoring and timely clinical decision-making [20], as exemplified by the EVIDENT II trial, which used a mobile app to monitor vital signs in high-risk cardiovascular patients [21]. These tools could reduce the need for face-to-face visits, facilitate monitoring, and enable the inclusion of vulnerable patients, or those with limited access to clinical trials [12]. These principles are pivotal to the decentralised clinical trial paradigm.

## Collaborative networks and support structures for investigator-initiated trials in primary care

In Spain, most investigator-initiated trials (IITs), referred to in national calls as non-commercial clinical research (NCCR), support a large proportion of clinical studies conducted in PC, primarily funded by competitive calls from the *Carlos III Health Institute* (ISCIII).

Networks such as the ISCIII Platform for Clinical Research – the Spanish Clinical Research Network (SCReN) – provide scientific, technical and administrative support for the comprehensive management of research projects. Specifically in the field of PC, SCReN is engaged in the active development of seven clinical studies, which are predominantly phase IV trials in cardiology, endocrinology, infectious diseases, pulmonology and neurology areas.

Several Results-Oriented Cooperative Research Networks in Health (RICORS), which belong to the ISCIII, are dedicated to PC research, primarily focusing on health promotion, disease prevention, education, collaborative research and dissemination of results, as implemented by recent studies [22]. These include the Primary Care Addiction Research Network (RIAPAd), the Research Network on Chronicity, Primary Care and Health Promotion (RICAPPS) and the former Network for Research in Preventive Activities and Health Promotion (REDIAPP, 2003–2021). Regional structures also play a pivotal role, with Madrid and Catalonia emerging as research leaders in Spain, driven by institutions such as the Jordi Gol i Gurina University Institute for Primary Health Care Research (IDIAPJGol, established in 1996) and the Foundation for Biosanitary Research and Innovation in Primary Care (FIIBAP, in 2016) in Madrid.

Other regions, such Andalusia, are also advancing the implementation of a consolidated framework for PC. The 2025 regional funding call includes a dedicated line for PC, district hospitals and high-resolution hospital centres, as well as a public–private innovation track, coordinated by Fundación Progreso y Salud (FPS). This initiative serves to integrate funding, governance, and research management.

**Figure 1.**
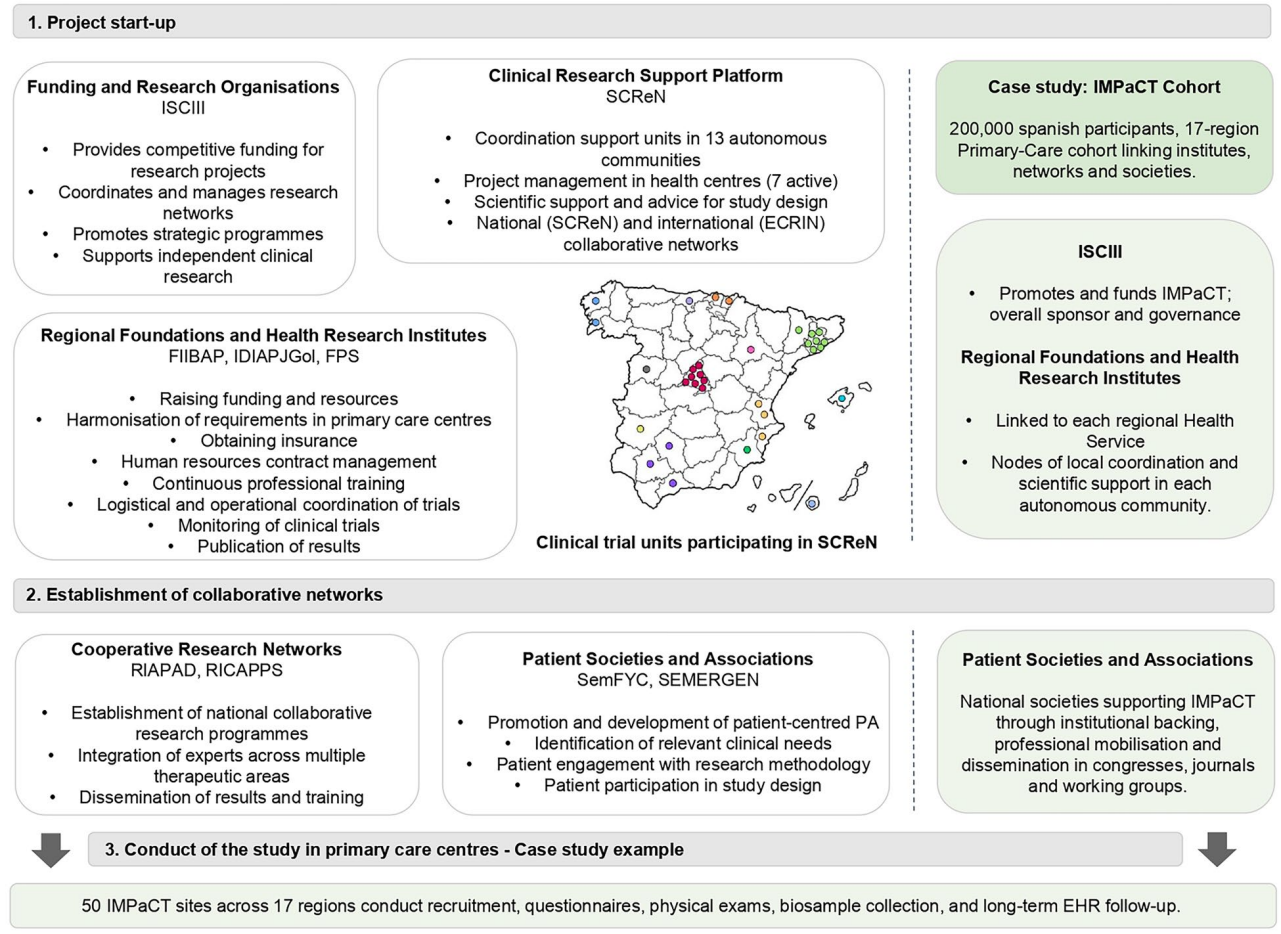
Schematic overview of the key actors and procedures involved in developing a clinical research project in primary care in Spain. A successful case study in Spain: IMPaCT Cohort, the Predictive Medicine program of Spain’s national precision medicine initiative led by ISCIII. It aligns national coordination, regional implementation structures, cooperative research networks and Primary Care scientific societies to implement a nationwide Primary-Care-based cohort, integrating recruitment, standardised phenotyping, biosampling/biobanking and long-term follow-up. ISCIII: Carlos III Health Institute; SCReN: Spanish Clinical Research Network; ECRIN: European Clinical Research Infrastructure Network; FIIBAP: Foundation for Biosanitary Research and Innovation in Primary Care; IDIAPJGol: Jordi Gol i Gurina University Institute for Primary Health Care Research; IMPaCT: Precision Medicine Infrastructure associated with Science Technology; FPS: Andalusian Public Foundation Progress and Health; RIAPAD: Primary Care Addiction Research Network; RICAPPS: Research Network on Chronicity, Primary Care and Health Promotion; SEMERGEN: Spanish Society of Primary Care Physicians; SemFYC: Spanish Society of Family and Community Medicine.

Ultimately, clinical research fundamentally depends on active patient collaboration. Patient associations and PC societies, such as the Spanish Society of Family and Community Medicine (SemFYC) and the Spanish Society of Primary Care Physicians (SEMERGEN), play a contributory role in establishing national collaborative programs and promoting patient- and community-centered research. Their active involvement enhances research designs, ensuring the relevance of the questions asked and the applicability of the results (Figure 1).

## Challenges and proposals for improvement in research

PC clinical research in Spain faces persistent, system-level constraints: limited funding, chronic staff shortages, and heavy clinical workloads that leave little protected time for research. The imbalance is stark: the ratio of family doctors is 0.8 per 1,000 inhabitants – less than half the hospital ratio of 1.9 – while nursing staffing shows a similar gap (0.7 versus 3.6 per 1,000) [2].

Previously identified barriers – pressure of care (mean 4.42/5), lack of time (4.36/5), insufficient methodological training (3.07/5) and limited institutional recognition (3.25/5) [23] – remain present. A 2022 systematic review in Ibero-American PC echoed training deficits – and, to a lesser extent, infrastructure – as recurrent obstacles [24]. Despite these conditions, professional motivation remains high, as seen in a 2017 national survey in which research training interest was rated 8.46/10 [25].

These pressures have tangible consequences: a recent meta-analysis estimates burnout at about one-quarter of Spanish PC physicians (24%) [26]. In parallel, many clinicians feel ill-prepared for regulatory and ethical procedures – contracting, ethics committee submissions, safety reporting–given limited prior experience. The combination of time constraints, training gaps, burnout, and scant recognition discourages participation in studies.

Creating a genuine research culture therefore requires structural reform rather than isolated initiatives. Spain needs university departments of Family and Community Medicine with stable resources, critical mass, and scientific leadership on a par with hospital units [27]. Embedded in universities and integrated with health services, such departments should articulate academic pathways (undergraduate, residency, continuing professional development), provide mentoring and internal seed calls, and host methodological platforms that support projects originating in health centres.

Legislation already offers partial tools. Spanish Law N° 17/2022 of September 5 enables up to 50% of the working day to be devoted to research through specific statutory categories or intensification schemes; in practice, however, application in PC remains exceptional. Access to funding is also perceived as poor (mean 3.44/10) [25]. Historically, only around 3–4% of AES-ISCIII (*Acción Estratégica en Salud* from ISCIII) research grants have been attributed to PC [28], although the 2024 AES-ISCIII call explicitly lists it as a priority area for the first time. Complementarily, the 2023 *Plan de Acción de Atención Primaria y Comunitaria* allocates €493 million to modernise facilities – an essential step to ensure that new and refurbished centres are research-ready.

To bridge these gaps, regional PC research foundations and collaborative networks – supported by universities – should provide continuous education, methodological mentoring, and regulatory guidance, helping practitioners design studies, and integrate research into daily workflows. A national coordinating hub linked to regional, university-anchored nodes – on the lines of DESAM-ForNet and PraksisNett – could accredit research-ready practices, provide on-site coordinators or research nurses, and deploy a federated, privacy-preserving IT backbone to run feasibility queries and enable EHR-embedded screening and recruitment. In parallel, following the NIHR Clinical Research Network (UK), practices should receive compensation or time buyout for research activity and gain access to service-support teams handling logistics, governance, and regulatory issue documentation. Structured training pathways – from modular ‘research-ready’ curricula to clinical-academic tracks – would sustain methodological and regulatory competence over time.

Public–private and inter-institutional collaboration can further expand funding, infrastructure, and training. Co-funded consortia in PC may facilitate earlier access to innovative medicines and improve quality of care. These partnerships can align with national R&D instruments – such as the *Proyectos en Colaboración Público-Privada of the Agencia Estatal de Investigación* under the 2024–2027 National R&D Plan, which mobilise over €560 million–and are reinforced by the revised Law N° 17/2022. At the regional level, PC research consortia that include universities and industry can secure financing, infrastructure, and knowledge transfer. Cross-level integration (PC–hospital) through joint research lines – chronic disease, guideline implementation, translational projects – can harness complementary datasets and incentives, embedding research in routine multidisciplinary care (Table 1).

Methodological challenges also require explicit attention. Low prevalence of certain clinical problems and high patient heterogeneity complicate case identification and the assembly of large, comparable samples. Recruiting general practitioners and patients across multiple centres, harmonising consent, follow-up, and data capture, demand substantial resources [18]. These constraints underscore the need for broad, interoperable networks linking health centres and registries across regions, supported by central methodological and logistical units. Combining routine EHR/registry data with prospective enrollment–enabling EHR-embedded screening and pooled recruitment – can increase efficiency and external validity.

## Conclusions and future prospects

Primary care should no longer be seen merely as a starting point for innovation but a cornerstone of evidence generation in real-world healthcare. Despite persistent barriers, heavy workloads, limited resources and regulatory complexity, its potential to deliver representative, practice-changing data remains vast. Digital platforms and interoperable EHR systems, combined with telemedicine and remote monitoring tools, help overcome time and logistical barriers by enabling automated data capture, centralised patient recruitment and continuous follow-up, thereby reducing administrative workload and broadening patient inclusion. Crucially, primary care’s close doctor–patient relationship and team-based context turn everyday encounters into practice-changing evidence where it matters most: real-world care. Public institutions and industry are increasingly investing in knowledge generation, while collaborative networks among professionals is laying the foundations for this. Consolidating this scientific basis for prevention and health is not aspirational but essential for improving population health.

Ensuring long-term sustainability requires embedding research within the health system through stable institutional frameworks, predictable funding, and protected research time. Spain can build on models such as the NIHR in the UK or DESAM-ForNet in Germany, adopting multi-level governance and hybrid funding to secure continuity. Dedicated national and regional funding, performance-based agreements, and capacity-building programs will be key to maintaining momentum.

Addressing these gaps now requires targeted, system-level action. Accordingly, policymakers, funders and professional societies should prioritise PC research by: (1) implementing interoperable registries across care levels; (2) protecting research time and support roles within PC teams; (3) ensuring sustained, targeted funding and minimum viable infrastructure; and (4) streamlining ethical and regulatory pathways for multicentre and decentralised trials. These steps would align research with everyday practice, enhance equity and external validity, and accelerate Spain’s capacity to generate practice-changing evidence from primary care.
